# Dr. John Bassett Chapin

**Published:** 1918-03

**Authors:** 


**Dr. John Bassett Chapin,** Jefferson Medical College, 1853, died in Chautauqua, age 88, Jan. 17. He first recommended changes in asylum construction to provide for the segregation of the insane in detached blocks or buildings according to classes and conditions. He wrote several monographs on the care and treatment of the insane.

